# Sea Anemone-Inspired Phase Change Composites for Efficient Heat Dissipation and Ultra-High Electromagnetic Interference Shielding

**DOI:** 10.34133/research.1075

**Published:** 2026-01-12

**Authors:** Xiaoling He, Wenjian Zhang, Tao Liu, Qianhui Lin, Zexi Zhang, Ying Chen, Jiye Luo, Chengqiang Cui, Xinxin Sheng

**Affiliations:** ^1^ State Key Laboratory of Precision Electronic Manufacturing Technology and Equipment, School of Electromechanical Engineering, Guangdong University of Technology, Guangzhou 510006, China.; ^2^ Guangdong Provincial Key Laboratory of Functional Soft Condensed Matter, School of Materials and Energy, Guangdong University of Technology, Guangzhou 510006, China.; ^3^ School of Chemical Engineering and Light Industry, Guangdong University of Technology, Guangzhou 510006, China.

## Abstract

Exigent demands for multifunctional composites integrating proficient thermal management and electromagnetic shielding functionalities arise directly from the accelerating sophistication and power intensification of modern electronic systems. Drawing inspiration from the efficient grasping tentacles of sea anemones, this study pioneers a bioinspired strategy for thermal and electromagnetic management in high-power electronics: architecting bioinspired carbon nanotubes (CNTs) interfaces on copper foam. Notably, the sea anemone tentacle-like CNTs architected on copper foam not only anchor abundant poly(styrene-ethylene-propylene-styrene)/n-docosane to enhance the composites’ latent heat capacity and leakage resistance but also interconnect with expanded graphite to establish multi-path thermal conduction networks while creating electromagnetic wave-reflecting heterogeneous interfaces. By leveraging CNTs as structural bridges, this design integrates the copper foam, expanded graphite, and polymer matrix into a continuous composite (CuFCE-2), achieving superb thermal conductivity (4.71 W/m·K). Consequently, CuFCE-2 excels in thermal management, suppressing chip temperatures by 60.6 °C (transient shock) and 15.7 °C (steady state). Critically, synergistic coordination across these bioinspired heterogeneous interfaces achieves prominent electromagnetic interference shielding, averaging 111.1 dB in the X-band (8.2 to 12.4 GHz). Collectively, the straightforward preparation method and exceptional properties of CuFCE-2 endow it with substantial application potential in electronics and communications, aerospace and defense, as well as new energy and energy storage systems.

## Introduction

In the tide of the intelligent era, the rapid evolution of communication technologies and the continuous advancement of computing power have driven human science and technology to an unprecedented pinnacle. However, the dramatic increase in chip heat flux and the escalating challenges of electromagnetic interference (EMI) have emerged as bottlenecks hindering technological implementation and product iteration [1]. For instance, insufficient heat dissipation can lead to elevated operating temperatures, ultimately resulting in performance degradation or even device failure [2,3]. Furthermore, excessive electromagnetic radiation not only compromises the stable operation of electronic systems but also poses potential risks to human health and information security [4]. Consequently, the development of multifunctional materials with both brilliant EMI shielding performance and efficient thermal management capabilities has emerged as a critical research priority for improving the safety and reliability of electronic devices [5,6].

It is well-established that the systematic optimization of nanomaterial design and arrangement within composites represents a highly effective strategy to concurrently enhance both thermal conductivity and the reliability of electromagnetic shielding [7,8]. In this regard, substantial research efforts have been dedicated to the microstructural design and innovation of various high-performance nanofillers, such as the development of novel nanostructures and the construction of diverse nanoscaffolds, aiming to achieve more robust or comprehensive performance advancements [9–11]. Among the diverse range of nanomaterials with varying morphologies and properties, carbon-based fillers, particularly carbon nanotubes (CNTs), stand out due to their intrinsic high thermal conductivity and exceptional electromagnetic shielding and microwave absorption capabilities, making them highly sought after for the development of high-performance thermal and electromagnetic shielding composites [12]. To address the challenges of CNTs aggregation and entanglement, which arise from their self-assembly tendencies and their exceptionally high aspect ratios, directed alignment of CNTs or the modification of other nanomaterials with CNTs has emerged as a predominant strategy. For instance, Liu et al. [13] utilized graphene sheets as the primary scaffold, interspersed with multi-walled CNTs, to form continuous thermal conduction pathways, thereby enhancing the out-of-plane thermal conductivity to 1.30 W/(m·K) while achieving an electromagnetic shielding effectiveness of 42 dB.

Recently, metal foams have gained attention as effective thermal scaffolds due to their high intrinsic thermal conductivity and self-supported, 3D interconnected porous structures. These characteristics facilitate efficient macroscopic heat transport while providing an ideal matrix for phase change material (PCM) encapsulation. For instance, Guo et al. [14] deposited a hybrid network of carbon fibers and multi-walled CNTs onto copper foam (CuF) to create a dual copper–carbon conductive framework. When infiltrated with paraffin, the resulting composite exhibited both high thermal conductivity and efficient solar-to-thermal conversion. Therefore, integrating thermally conductive fillers into metal-foam scaffolds, followed by PCM encapsulation, offers a promising approach for developing multifunctional composites that combine heat dissipation with thermal energy storage.

In this thesis, a multi-network phase change composite (CuFCE-2) was fabricated using expanded graphite (EG) as the microwave-absorbing and thermally conductive filler, CuF decorated with in situ grown CNTs (CuF-CNTs) as the structural framework, and a mixture of hydrogenated version poly(styrene-ethylene-propylene-styrene) (SEPS) and n-docosane (C22) as the organic matrix. Like sea anemone tentacles, CNTs grow on CuF’s surface, providing abundant contact sites that facilitate efficiently dual heat/electrical conduction. Together, the elastic polymer network formed by SEPS/C22, the bridging network enabled by EG, and the dual-skeleton structure of CuF-CNTs construct a robust multi-network architecture, which effectively suppresses PCM leakage. As a result, CuFCE-2 exhibits admirable thermal conductivity (4.71 W/m·K), high electrical conductivity (4.29 × 10^6^ S/m), and strong light absorption (absorbance of 11.2), endowing the composite with superior chip thermal management, outstanding EMI shielding, and efficient electrothermal/photothermal conversion performance. In conclusion, the multifunctional phase change composites developed herein offer effective thermal management and EMI shielding, providing a practical solution to the dual challenges of heat accumulation and electromagnetic pollution in high-frequency electronic devices.

## Results and Discussion

**Fig. 1.**
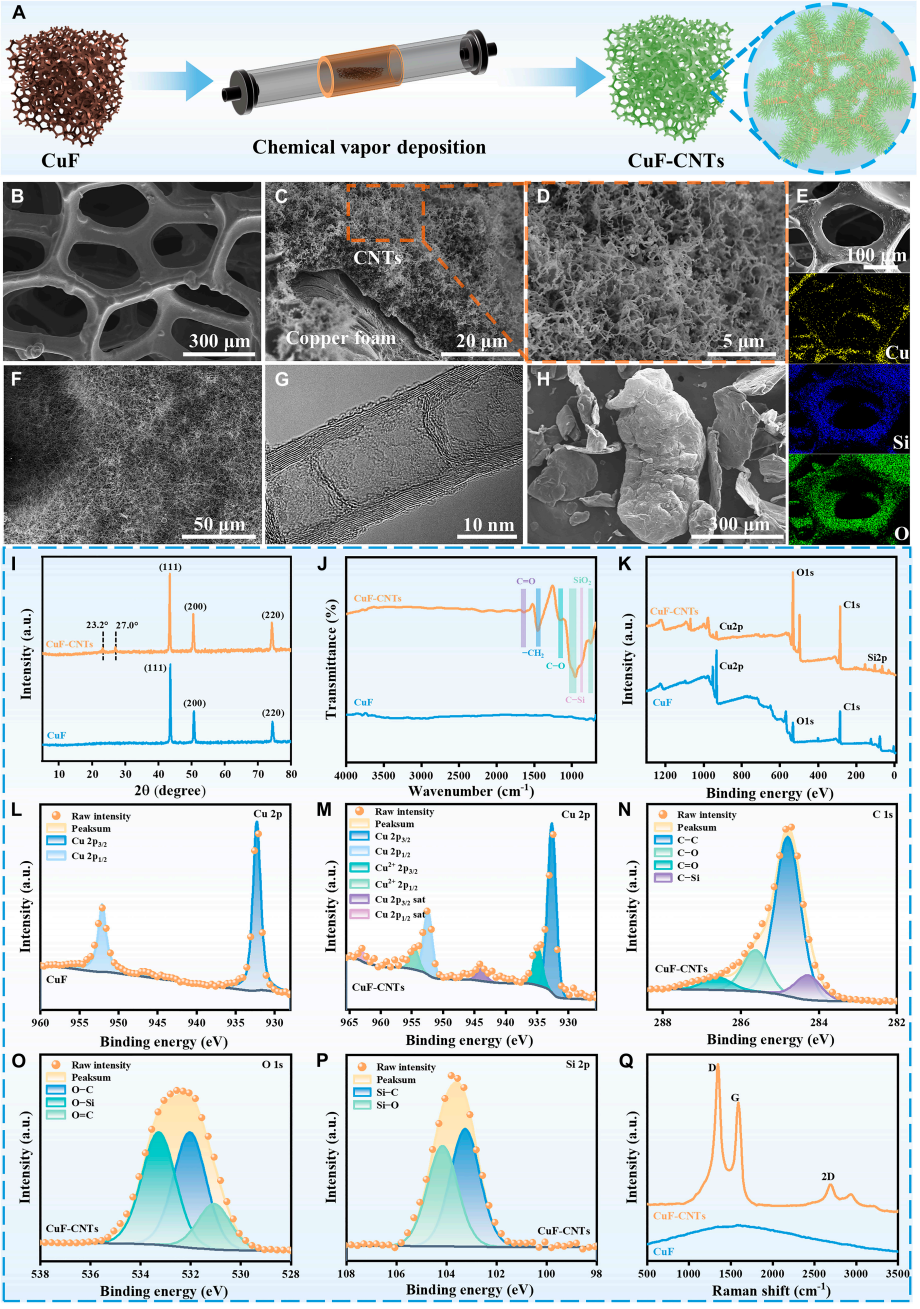
Preparation and physicochemical characterization of CuF-CNTs. (A) Schematic illustration of the preparation process of the CuF-CNTs. FESEM of (B) CuF and (C and F) CuF-CNTs. (D) is a magnified view of (C). (E) EDS mapping of F-CuF. (G) TEM of CNTs. (H) FESEM of EG. (I) XRD curves. (J) FTIR curves. (K) XPS wide-scan spectrum. (L) Cu 2p spectrum of CuF. (M) Cu 2p spectrum of CuF-CNTs. (N) C 1s spectrum of CuF-CNTs. (O) O 1s spectrum of CuF-CNTs. (P) Si 2p spectrum of CuF-CNTs. (Q) Raman spectrum.

As depicted in Fig. 1A, CNTs were grown on CuF through chemical vapor deposition to construct a thermally conductive CuF-CNTs framework with sea anemone-like structure. Subsequently, C22, SEPS, and EG were thoroughly mixed and infused into the CuF-CNTs framework through vacuum impregnation and hot pressing, resulting in the final CuFCE-2 composite. To gain in-depth understanding of the composite material, a comprehensive characterization protocol was systematically implemented, encompassing morphological analysis, physicochemical property assessment, and structural stability evaluation, alongside functional performance tests including thermal management metrics, electromagnetic shielding effectiveness, and energy conversion efficiency. Critically, computational thermal modeling (COMSOL Inc., Los Angeles, CA) was implemented to validate experimental findings, enabling predictive analysis of the composite’s thermal regulation performance under operational conditions.

### Characterization of CuF-CNTs

The detailed microstructure and morphology of CuF-CNTs at various stages of synthesis were analyzed using field-emission scanning electron microscopy (FESEM), energy-dispersive spectroscopy (EDS), and transmission electron microscopy (TEM). The surface of the pristine CuF is relatively smooth and exhibits a porous, 3D continuous network structure, along with a certain capacity for C22 adsorption (Fig. 1B). After silanization modification, a rough texture appeared on the CuF surface, resulting from the formation of a silicon oxide compound layer (Fig. 1E), which promotes the subsequent growth of CNTs. As shown in Fig. 1C, D, and F, high-density CNTs were successfully grown on the CuF surface by chemical vapor deposition, forming a structure similar to anemones. The CNTs exhibited a multi-walled structure with an average diameter of approximately 25 nm (Fig. 1G). Apparently, the in situ-grown high-density CNTs fully cover the CuF surface, greatly increasing its specific surface area to construct an efficient thermal conduction network. Moreover, EG with a worm-like morphology was introduced as a thermal conductive filler (Fig. 1H), which was beneficial for further contact with CNTs and strengthened the thermal conductive network.

Structural stability is crucial for material applications, especially in demanding operating environments where material failure directly impacts device functionality [15]. Therefore, we evaluated the structural stability of CuF-CNTs under 2 conditions: stirring at 500 rpm (Fig. S1A) and high-power ultrasonication at 160 W (Fig. S1F). As shown in Fig. S1D, no notable change was observed in the ethanol solution after 8 h of stirring with the CuF-CNTs. After filtration, only a small amount of CNTs detachment was detected on the filter paper (Fig. S1E), primarily from the initial stage of stirring (1 h), which is attributed to a minor presence of loosely bound CNTs on the CuF surface. FESEM observations of CuF-CNTs after 8 h of stirring revealed that a large number of CNTs remained on the CuF surface, confirming the splendid structural stability of CNTs on the substrate (Fig. S1B and C). Similarly, under high-power ultrasonication at 160 W, CuF-CNTs maintained their structural integrity, showing the same excellent stability as under stirring conditions (Fig. S1H). The CNTs on the CuF surface can enhance the capillary effect, and combined with the adsorption capacity of EG, this is beneficial for preventing PCM leakage [16,17].

To further investigate the physicochemical properties of CuF-CNTs, the original CuF and CuF-CNTs were characterized using x-ray diffraction (XRD), Fourier transform infrared (FTIR), x-ray photoelectron spectroscopy (XPS), and Raman spectroscopy. Firstly, their crystal structures and phase compositions were examined by XRD, as shown in Fig. 1I. In the XRD pattern of pristine CuF, the diffraction peaks at 2θ = 43.5°, 50.7°, and 74.4° correspond to the (111), (200), and (220) crystal planes of copper, respectively [18]. For CuF-CNTs, new diffraction peaks appeared at 2θ = 23.2° and 27°. The peak at 23.2° corresponds to amorphous silicon–oxygen compounds, while the peak at 27° is attributed to the (002) crystal plane of the graphitic structure in CNTs, confirming the successful growth of CNTs on CuF following silanization modification [19].

The chemical structure of CuF-CNTs was analyzed using FTIR and XPS. As shown in Fig. 1J, the FTIR spectrum of pristine CuF exhibits almost no absorption peaks, indicating its composition as pure copper. In contrast, the CuF-CNTs spectrum displays characteristic bands at 1,640, 1,450, and 1,152 cm^−1^, corresponding to the C=O stretching vibration, the –CH_2_ deformation and in-plane rocking vibrations, and the C–O stretching vibration, respectively [20,21]. In addition, the stretching vibration of Si–O–Si (1,026 cm^−1^) and the bending and symmetric stretching vibrations of Si–O–Si chains (750 cm^−1^) were observed in the CuF-CNTs spectrum, which are attributed to the formation of silicon oxide compounds through the hydrolysis of tetraethyl silicate (TEOS) and 3-aminopropyltriethoxysilane (APTES) during the silanization process [22]. Moreover, the C–Si stretching vibration (850 cm^−1^) observed in the CuF-CNTs spectrum further confirms that the CNTs are anchored to the CuF surface by a silicon oxide compound transition layer [23].

In Fig. 1K, the C 1s peak in the XPS spectrum of CuF-CNTs is significantly enhanced compared to that of pristine CuF, indicating the successful introduction of CNTs. Additionally, the increased intensity of the O 1s peak and the emergence of a new Si 2p peak further confirm the silanization of CuF. In the Cu 2p spectrum of pristine CuF, strong peaks are observed at 932.3 and 952.1 eV, corresponding to Cu 2p^3/2^ and Cu 2p^1/2^, respectively (Fig. 1L). In contrast, the Cu 2p spectrum of CuF-CNTs (Fig. 1M) shows additional peaks at 934.8 eV (Cu^2+^ 2p^3/2^), 954.5 eV (Cu^2+^ 2p^1/2^), 943.8 eV (Cu 2p^3/2^ sat), and 962.9 eV (Cu 2p^1/2^ sat), which are likely due to partial oxidation of CuF during the silanization process [24]. After the growth of CNTs, the C 1s spectrum of CuF-CNTs was deconvoluted into 4 components: C–C, C–O, C=O, and C–Si (Fig. 1N) [25,26]. Similarly, the O 1s spectrum was deconvoluted into 3 components: O–C, O–Si, and O=C (Fig. 1O) [27]. These results confirm the presence of oxygen-containing functional groups and silicon–oxygen compounds formed during the CuF surface modification process. The Si 2p spectrum also reveals distinct peaks at 103.2 and 104.1 eV, corresponding to Si–C and Si–O bonds, respectively (Fig. 1P), suggesting that CuF and CNTs may be connected through C–Si–O covalent bonds [28].

The Raman spectrum of CuF-CNTs (Fig. 1Q) exhibits distinct D, G, and 2D peaks at 1,350, 1,590, and 2,700 cm^−1^, respectively. The presence of these peaks, absent in pristine CuF, confirms the successful synthesis of CuF-CNTs. The ratio of the D peak to G peak intensity (*I*_D_/*I*_G_) is commonly used to assess the degree of disorder in carbon materials [29]. For CuF-CNTs, the *I*_D_/*I*_G_ value is 1.35, indicating a relatively high level of structural disorder. This increased disorder is primarily attributed to the simultaneous formation of amorphous carbon during the high-temperature chemical vapor deposition process [30]. In summary, multi-scale physical and chemical characterizations collectively confirm the successful formation of CuF-CNTs skeleton.

### Characterization of composites

**Fig. 2.**
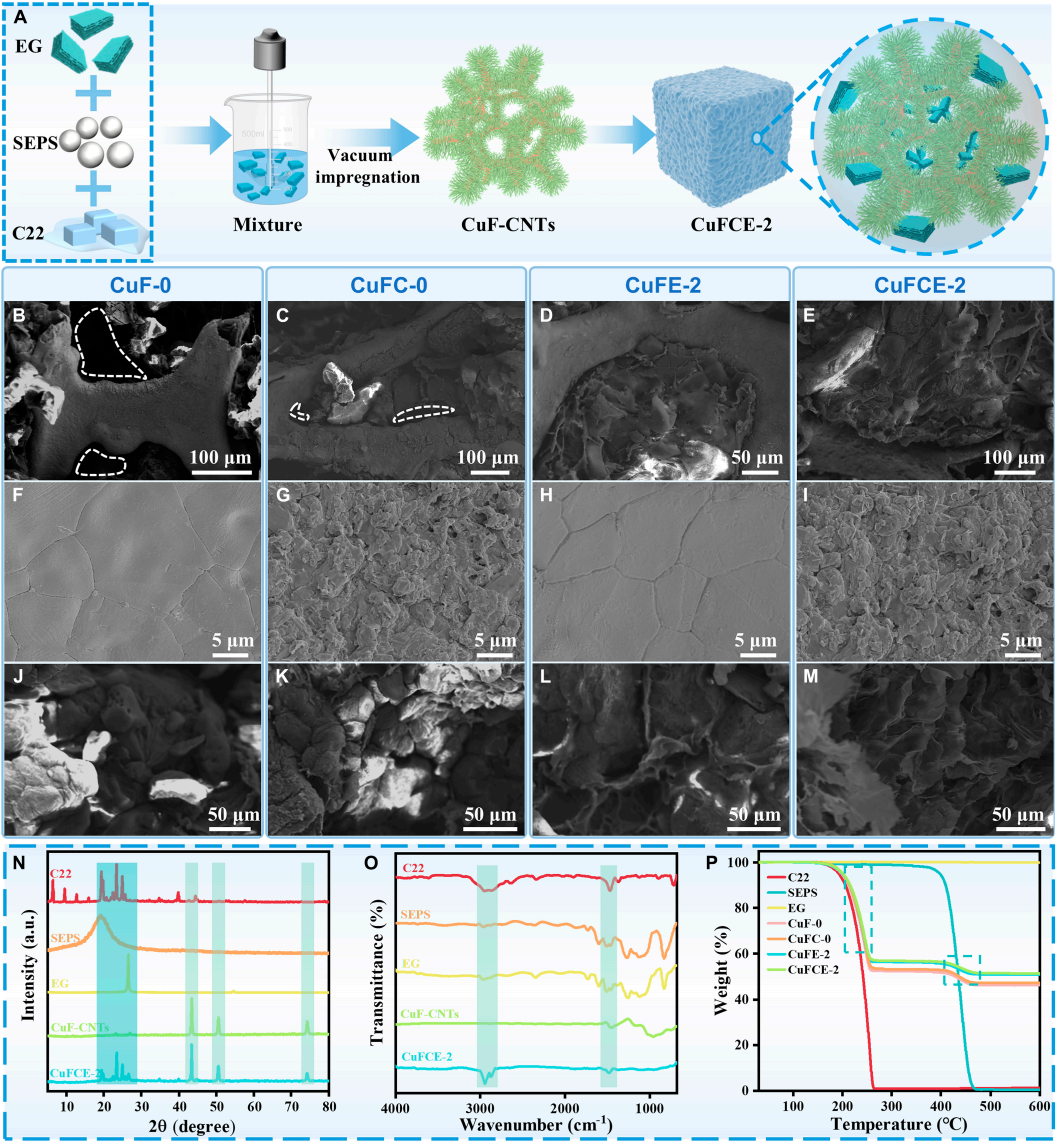
Preparation and physicochemical characterization of phase change composites. (A) Schematic illustration of the preparation process of CuFCE-2. FESEM of (B) CuF-0, (C) CuFC-0, (D) CuFE-2, and (E) CuFCE-2. FESEM of the metal skeletons in (F) CuF-0, (G) CuFC-0, (H) CuFE-2, and (I) CuFCE-2. FESEM of the matrix in (J) CuF-0, (K) CuFC-0, (L) CuFE-2, and (M) CuFCE-2. (N) XRD curve. (O) FTIR curve. (P) thermogravimetric analyzer (TGA) curve.

As shown in Fig. 2A, phase change composites were fabricated using the above-synthesized CuF-CNTs as the supporting framework. The composite’s shape stability was evaluated separately at 60 and 80 °C (Fig. S2A and B). The results show that SEPS exhibits a certain encapsulation effect on C22 due to their similar chain structures and good compatibility, which allows liquid C22 molecules to penetrate into the intermolecular spaces of SEPS and become locked within the polymer matrix [31]. Notably, all composites exhibited admirable shape stability with no PCM leakage. This is primarily attributed to the synergistic effect of multiple structural networks that effectively encapsulate C22, including the physical barrier formed by SEPS, the spatial confinement provided by the metal skeleton, the dual blockade and adsorption from the microcavities of high-density CNTs, and the strong adsorption capability of EG.

The performance of the composite is closely related to its microstructure. As illustrated in Fig. 2B, obvious pores are present in CuF-0, caused by the leakage of PCM during hot pressing. After the growth of CNTs on CuF, the number of pores in the CuFC-0 composite was significantly reduced (Fig. 2C), which is ascribed to the microcavities formed by the high-density CNTs that can adsorb and retain C22, thereby effectively reducing PCM leakage. According to Fig. 2F to I, observation of the metal framework within the composite reveals that the CNT-modified CuF is coated with the polymer matrix (SEPS/C22), indicating that the incorporation of CNTs enhances the interfacial compatibility between the CuF and the polymer phase. With the addition of 2 wt % EG, no pores were observed in the cross-sections of CuFE-2 and CuFCE-2 (Fig. 2D and E). Furthermore, the mixture was in full contact with the metal framework, forming a dense and continuous corrugated structure (Fig. 2L and M). This demonstrates that EG not only enhanced the adsorption capacity for C22 but also optimized the heat transfer pathways.

The crystal structure, chemical composition, and thermal stability of the composite and its raw materials were analyzed by XRD, FTIR, and TGA. As shown in Fig. 2N, the XRD pattern of CuFCE-2 exhibits multiple strong diffraction peaks at 19.5°, 22.4°, 23.5°, 25°, and 26.4°, corresponding to the raw materials C22, SEPS, and EG. Notably, the diffraction peak of EG at 2θ = 26.4° is strong and sharp, indicating a highly ordered graphite structure. This provides the conditions for EG to form a 3D interconnected network within the composite, establishing efficient phonon transport [32]. Simultaneously, the diffraction peaks at 2θ = 43.5°, 50.7°, and 74.4° correspond to the CuF skeleton. The presence of characteristic peaks from all raw materials in the XRD spectrum of CuFCE-2 indicates that the CuF-CNTs framework successfully adsorbed the mixture without altering the crystal structure of C22.

Moreover, the FTIR spectrum of C22 exhibited 3 main peaks at 2,946, 2,870, and 1,460 cm^−1^, corresponding to the asymmetric stretching vibration of –CH_3_, the symmetric stretching vibration of –CH_2_–, and the deformation and in-plane rocking vibrations of –CH_2_, respectively (Fig. 2O) [31]. These peaks are also present in the CuFCE-2 composite, with no new absorption bands observed. This reveals that the raw materials are physically mixed and adsorbed within the composite, and no chemical reactions occur during the process. In Fig. 2P, the composite exhibits 2 distinct weight loss stages: the decomposition of C22 (180 to 270 °C) and the decomposition of SEPS (380 to 470 °C). EG shows negligible weight loss between 30 and 600 °C, so the remaining residue in the composite consists of the metal skeleton and EG. Furthermore, the composite demonstrates remarkable thermal stability within the working temperature range of 30 to 150 °C.

### Thermal management performance

**Fig. 3.**
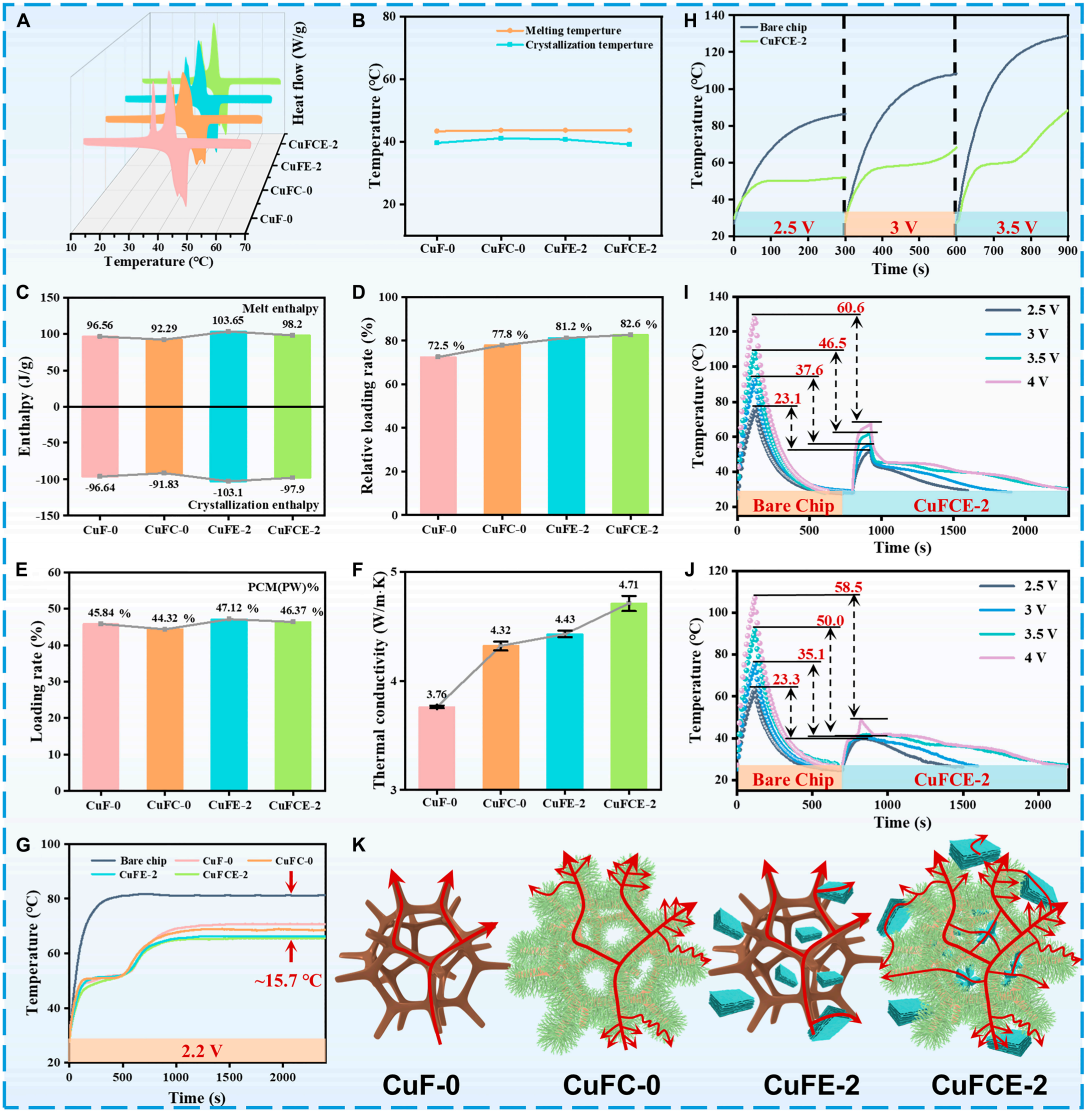
(A) DSC curve. (B) Phase change temperatures. (C) Melting enthalpy and crystallization enthalpy. (D) SEPS/C22/EG relative loading rates. (E) PCM (PW) loading rates. (F) Thermal conductivity. Temperature change curves of the simulated chip during (G) 40 min and (H) 300 s of operation. Operating temperature change curves of the (I) simulated chip and (J) sample after 120 s of operation. (K) Schematic illustration of thermal conduction.

Heat storage capacity is one of the most important characteristics of phase change composites. We used differential scanning calorimetry (DSC) to evaluate the phase change behavior and thermal properties of the composites. Figure 3A shows the DSC curves of the 4 composites, which overlap considerably, with very similar phase transition temperatures (Fig. 3B). This indicates that the presence of the metal skeleton, SEPS, CNTs, and EG does not affect the phase transition behavior of C22. Figure 3C presents the melting and crystallization enthalpies of the composites. CuFE-2 exhibits higher enthalpy values than the other samples, attributed to the strong adsorption of EG on the PCM, indicating its superior heat storage capacity. The PCM loading in CuFCE-2 was slightly lower than that in CuFE-2, which may be attributed to the growth of a large number of CNTs occupying the space of the polymer matrix. Nevertheless, the mixture (SEPS/C22/EG) achieved a high loading factor of 82.6% in CuFCE-2 (Fig. 3D), revealing minimal structural defects [33]. The high thermal conductivity of CuFCE-2, reaching 4.71 W/m·K as depicted in Fig. 3F, confirms this. In addition, the prominent thermal conductivity of CuFCE-2 can also be attributed to the multiple heat conduction pathways formed among CuF, CNTs, and EG [34].

To evaluate the electronic thermal management performance of CuFCE-2, an experimental platform (Fig. S3) simulating chip operation was constructed [35]. Figure 3G illustrates that the temperature of the bare simulated chip rapidly rose to 80 °C and stabilized within 400 s. In contrast, when the composites were applied, the chip’s temperature increased more gradually, taking 1,200 s to reach a stable state. This demonstrates that the composite effectively slows the rate of temperature rise, helping to prevent sudden overheating during operation. After reaching a stable state, the steady-state temperature of the chip with the CuFCE-2 composite is only 65.5 °C, which is approximately 15.7 °C lower than the operating temperature of the bare chip. This significant reduction is attributed to the combined effects of its high thermal conductivity and excellent enthalpy, fully confirming the outstanding efficiency of CuFCE-2 in long-term thermal management applications for electronic chips. With a further increase in the output voltage of the simulated chip (Fig. 3H), the temperature difference between the chip protected by CuFCE-2 and the bare chip became more pronounced as heat generation intensified. This suggests that CuFCE-2 can rapidly absorb the heat released from the chip, providing an effective thermal buffering effect under high heat flux conditions.

To further evaluate the thermal management performance of CuFCE-2 under high thermal shock conditions, the temperature changes of the simulated chip (Fig. 3I) and the sample surface (Fig. 3J) were recorded separately. After 120 s of exposure to different high-voltage outputs, the temperature differences between the chip loaded with CuFCE-2 and the bare chip were 23.1 °C (2.5 V), 37.6 °C (3 V), 46.5 °C (3.5 V), and 60.6 °C (4 V). As the output voltage increases, CuFCE-2’s effectiveness in mitigating transient thermal shock is further enhanced, highlighting its brilliant heat absorption performance. Remarkably, CuFCE-2 lowered the simulated chip’s temperature by 60.6 °C at 4 V, showcasing robust protection against transient thermal shock, which is an invaluable advantage for electronics facing rapid and intense heat bursts. The superior thermal management performance of CuFCE-2 originates from its dual-mode mechanism: rapid heat absorption in PCM and efficient heat dissipation through thermally conductive pathways. In summary, comprehensive chip thermal management tests have certificated both the superb thermal conductivity of CuFCE-2 and its high efficiency in thermal management applications.

**Fig. 4.**
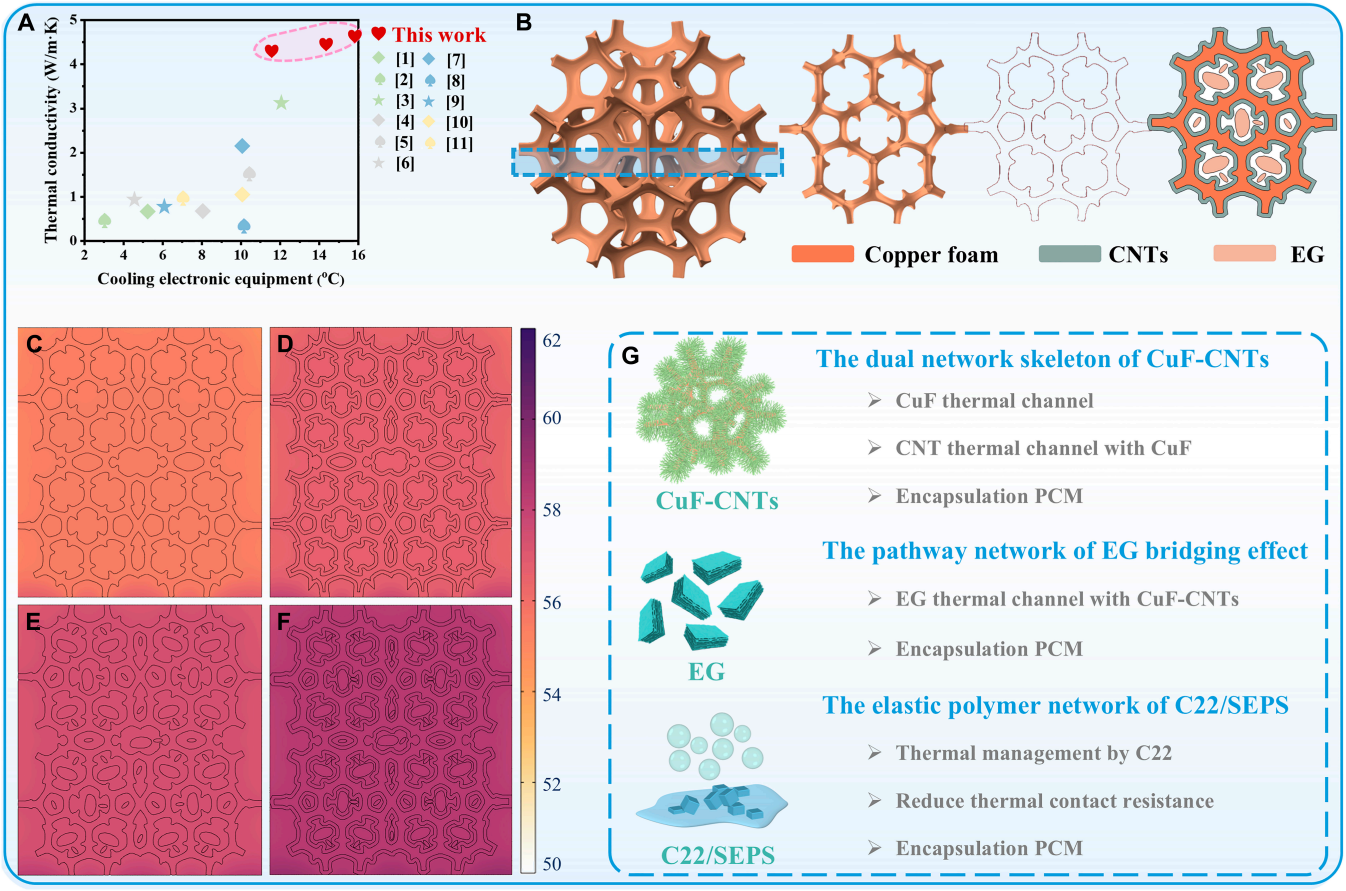
(A) Comparison of thermal conductivity and cooling capacity between this work and reported phase change composites. (B) Schematic illustration of the 2D geometric model construction for CuFCE-2. Finite element simulation results of (C) CuF-0, (D) CuFC-0, (E) CuFE-2, and (F) CuFCE-2. (G) Schematic diagram of thermal management mechanism.

The CuFCE-2 phase change composite demonstrates exceptional thermal conductivity and thermal management capabilities (Fig. 4A). To further elucidate its heat transfer mechanism, a representative cross-section was selected following 3D reconstruction of the CuF scaffold, which was subsequently populated with fillers and matrix materials. A phase change heat transfer solid–fluid coupling model was developed via finite element analysis to simulate the thermal behavior of the composite, with results presented in Fig. 4B. The simulation results are in excellent agreement with the experimental data, with all deviations within 1 °C (Fig. S4). Under identical heating power and temperature conditions, CuFCE-2 exhibits superior thermal diffusion kinetics, rapidly distributing thermal energy throughout the composite matrix, outperforming comparative samples in heat transfer efficiency (Fig. 4F). The splendid thermal management performance of CuFCE-2 originates from synergistic contributions of synthesized CuF-CNTs heterostructures, cooperative effects between CNTs and EG, flexible interface enabled by SEPS, and phase change-driven thermal energy storage from C22.

Primarily, densely packed CNTs grown on the CuF skeleton establish continuous heterogeneous interfaces and anemone tentacle-like architectures, simultaneously enhancing interfacial bonding strength and thermal pathway density [36]. The resulting 3D heterostructure effectively regulates phonon scattering at the interface, enabling efficient transfer of phonon vibrational energy and thereby improving thermal conductivity [37]. Subsequently, the individual introduction of CNTs and EG builds thermal bridges between the metal skeletons and forms independent heat-conducting pathways. CNTs markedly increase the specific surface area of the metal framework, providing abundant high-density thermal contact points that facilitate synergistic interactions with EG and CuF to form multidimensional thermal conduction networks for efficient heat dissipation from the chip (Fig. 3K). Furthermore, SEPS imparts a certain degree of flexibility to the C22/SEPS/EG mixture, which effectively reduces the contact thermal resistance caused by interfacial mismatch in practical applications. Finally, the multi-level encapsulation network formed by SEPS, CNTs, EG, and the metal skeleton effectively prevents C22 leakage, allowing its latent heat storage capacity to be fully utilized for temperature regulation. Therefore, CuFCE-2 integrates multiple synergistic thermal optimization strategies, resulting in admirable thermal management performance (Fig. 4G).

### EMI shielding properties

**Fig. 5.**
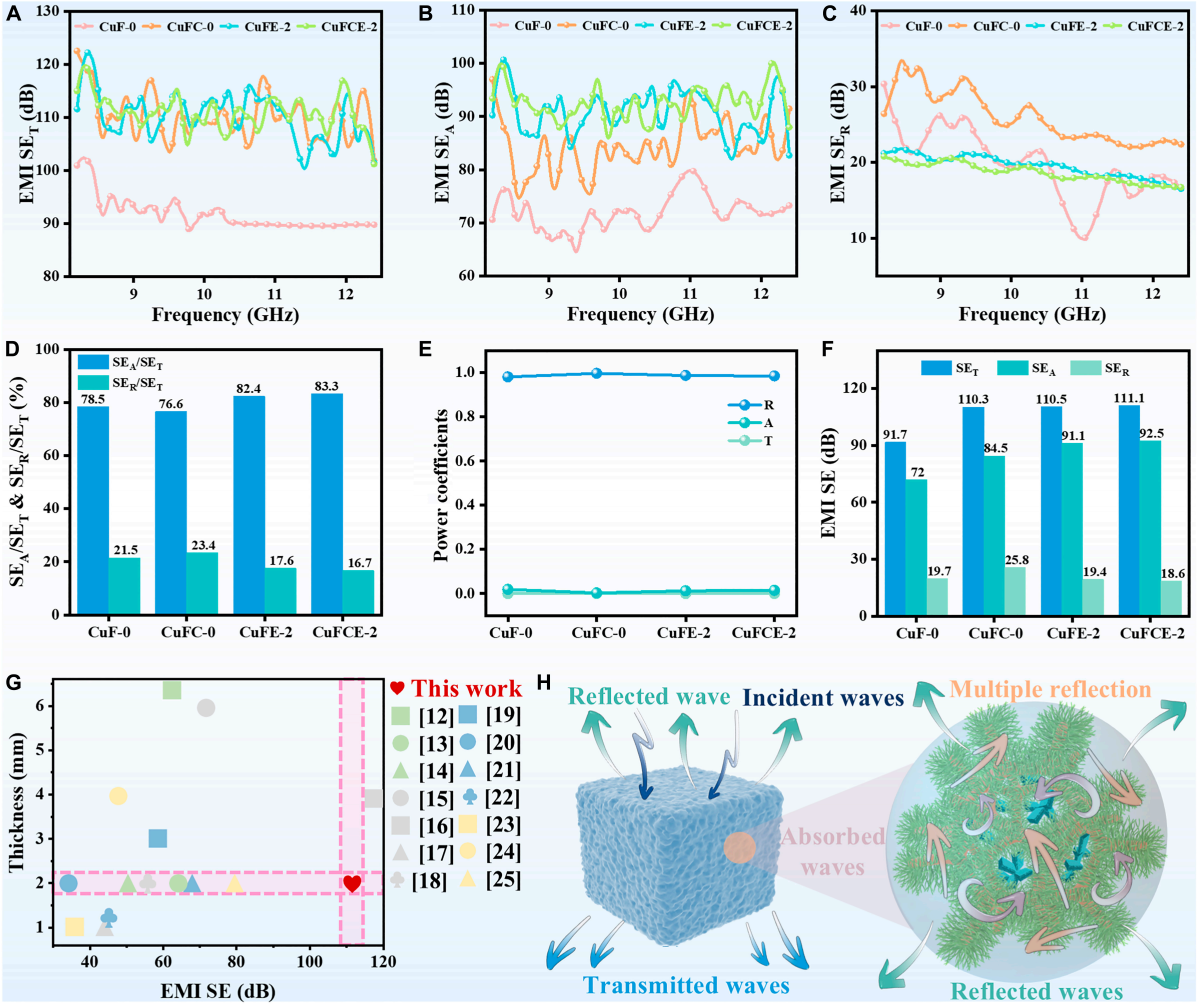
(A) SE_T_ of the samples. (B) SE_A_ of the samples. (C) SE_R_ of the samples. (D) Ratios of SE_A_/SE_T_ and SE_R_/SE_T_. (E) R, A, and transmission coefficient of samples. (F) Average SE_T_, SE_A_, and SE_R_ of samples. (G) Comparison of thickness and EMI capability between this work and reported composite materials. (H) Electromagnetic shielding mechanism of CuFCE-2.

The guided wave method was used to evaluate the EMI shielding effectiveness of the composites within the frequency range of 8.2 to 12.4 GHz. The total shielding effectiveness (SE_T_) comprises 3 components: reflection shielding (SE_R_), absorption shielding (SE_A_), and multiple reflection shielding (SE_M_) [38]. With the growth of CNTs and the incorporation of EG, the SE_T_ and SE_A_ of CuFCE-2 increased remarkably, while the SE_R_ exhibited a decreasing trend (Fig. 5A to C). This shift indicates that the synergistic effect of CNTs and EG effectively modulates the propagation path and loss mechanisms of electromagnetic waves within the composite, thereby greatly raising its electromagnetic wave absorption capability (Fig. 5D). The increase in the SE_A_/SE_T_ ratio heightens the material’s ability to dissipate electromagnetic waves and reduces secondary electromagnetic pollution caused by reflection [39]. However, for EMI shielding materials that are predominantly absorption-based, the SE_R_ is generally maintained below 3 dB [40]. Obviously, the reflection coefficient (*R*) of all samples exceeds 90% in Fig. 5E, while the absorption coefficient (*A*) is especially lower, which reveals that the majority of incident electromagnetic waves are still reflected by the surface of the material, with only a small portion of the energy being effectively absorbed. Therefore, reflection remains the dominant mechanism in the electromagnetic shielding process of this material system and is primarily responsible for its shielding effectiveness. Particularly, the average SE_T_ value of CuFCE-2 achieved an impressive 111.1 dB (Fig. 5F), meeting and exceeding the standard requirement (>100 dB) for military-grade shielded enclosures, demonstrating its considerable potential for EMI shielding applications (Fig. 5G).

As illustrated in Fig. 5H, when electromagnetic waves strike the surface of the material, a remarkable impedance mismatch between the air and the composite causes the majority of the waves to be reflected, providing an initial level of shielding [41]. Subsequently, the portion of the wave that penetrates the material encounters numerous heterogeneous interfaces within the multi-network structure [42,43]. These interfaces induce strong interfacial polarization, electric dipole polarization, and conductive losses, thereby effectively suppressing EMI [44]. Furthermore, the sea anemone bionic structure of CuF-CNTs primarily induces natural resonance-based magnetic loss and conduction loss, effectively attenuating EMI energy [45]. Specifically, the high-density CNTs tentacle arrays within this anemone-mimetic architecture create numerous microcavities that facilitate multi-reflection and absorption of electromagnetic waves, thereby prolonging propagation pathways and boosting energy dissipation efficiency [46]. Simultaneously, the CNTs tentacles anchor dispersed EG, establishing additional conductive pathways that induce significant interfacial conduction losses, collectively enhancing the overall electromagnetic shielding effectiveness. The elastic polymer network of C22/SEPS both refines the pore size within the CuF-CNTs skeleton to prevent electromagnetic wave leakage and significantly contributes to polarization loss. The pathway network formed by the EG bridging effect facilitates multiple reflections and absorption of electromagnetic waves, thereby extending their propagation path and enhancing energy dissipation efficiency [47].

### Multi-source-driven conversion performance

**Fig. 6.**
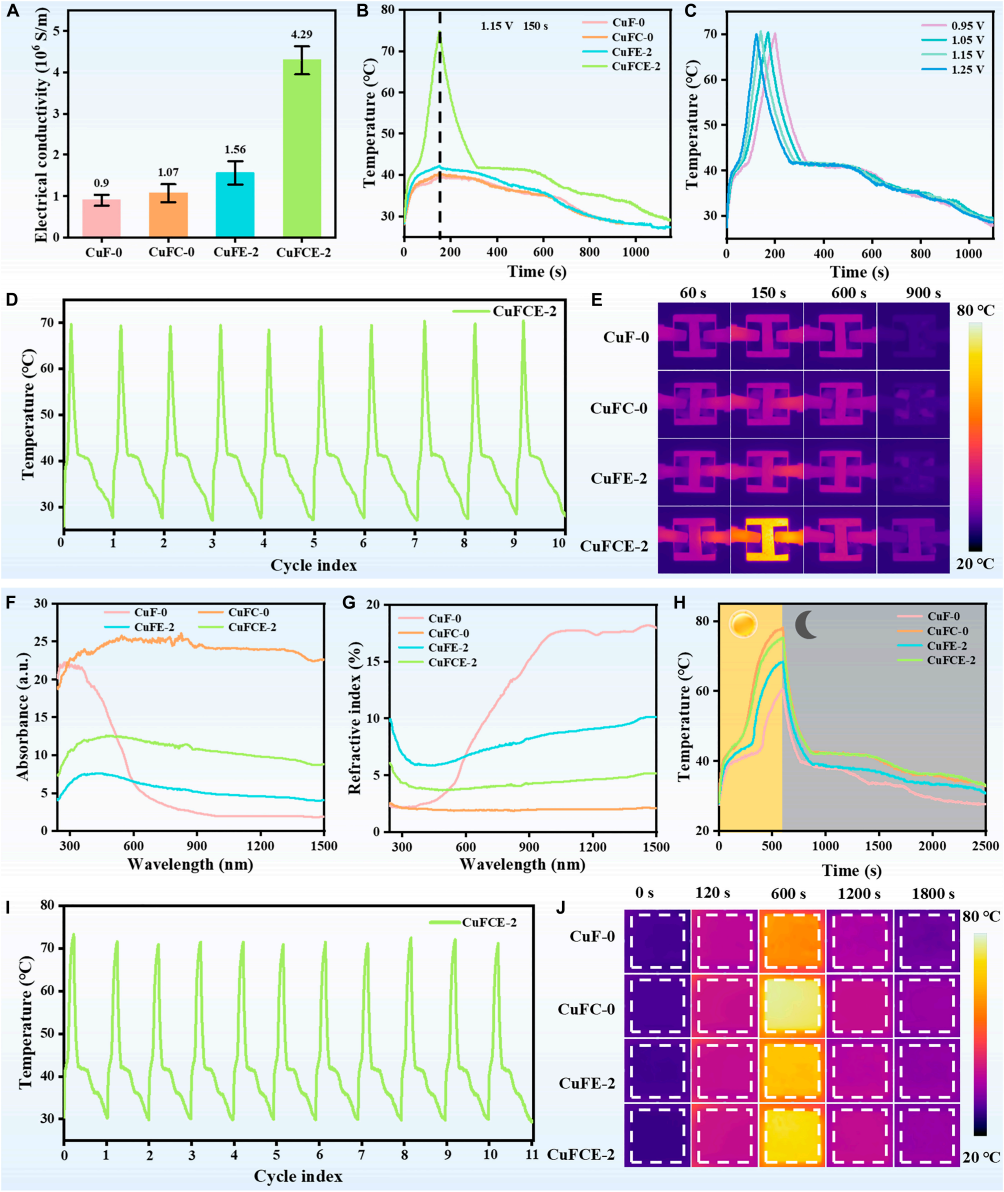
(A) Electrical conductivity. (B) Temperature change curve of specimens during electrothermal conversion at 1.15 V for 150 s. (C) Temperature variation curve of CuFCE-2. (D) Electrothermal cycle curve. (E) Thermal infrared image of the sample during electrothermal conversion under the conditions of 1.15 V and 150 s. (F) Absorbance spectra. (G) Reflectivity spectra. (H) Temperature variation curves of composites over time at a light intensity of 160 mW/cm^2^. (I) Photothermal cycle curves of CuFCE-2 at a light intensity of 160 mW/cm^2^. (J) Thermal images of samples captured with an infrared thermal image at a light intensity of 160 mW/cm^2^.

The electrical conductivity of a material directly influences its electrothermal conversion performance. As shown in Fig. 6A, the conductivity of the composites was evaluated. Benefiting from the presence of the metal skeleton, CuF-0 exhibits a conductivity of 0.9 × 10^6^ S/m. The conductivity was further enhanced by the in situ growth of CNTs and the addition of EG, both of which are highly conductive materials. Specifically, CuFC-0 and CuFE-2 achieve conductivities of 1.07 × 10^6^ S/m and 1.56 × 10^6^ S/m, respectively. It is worth noting that the conductivity of CuFCE-2 was significantly increased to 4.29 × 10^6^ S/m. This improvement is attributed to the synergistic effect of CNTs and EG, which together form a more continuous and integrated conductive network with the metal skeleton, thereby reducing contact resistance and improving overall conductivity.

Joule’s law is the fundamental principle governing electrothermal conversion. By regulating voltage and duration, one can effectively assess a material’s ability to generate Joule heat. To evaluate the electrothermal conversion performance of the composites, each sample was subjected to electrical input, and the resulting temperature changes were monitored using a thermal infrared camera (FLIR, Fig. 6E). After 150 s of power supply at 1.15 V, CuFCE-2 rapidly surpassed the phase change process and reached a temperature of 74.8 °C (Fig. 6B), due to its ultra-high conductivity. In contrast, the other 3 samples were just beginning to undergo phase change, with temperatures of 42.2 °C (CuFE-2), 40.3 °C (CuFC-0), and 39.5 °C (CuF-0), which aligns well with the conductivity test results. The electrothermal conversion performance of CuFCE-2 was further investigated at different voltages (Fig. 6C). As the voltage increased, the time required to reach 70 °C decreased from 200 s (0.95 V) to 170 s (1.05 V), 140 s (1.15 V), and 120 s (1.25 V). These results prove that CuFCE-2 can heat up rapidly even at low voltages, highlighting its fast thermal response and outstanding electrothermal conversion performance [48]. Moreover, the nearly identical pulse heating cycle curves over 10 cycles (Fig. 6D) demonstrate the exceptional electrothermal conversion stability of CuFCE-2. In conclusion, the ultra-high electrical and thermal conductivity of CuFCE-2 enables it to deliver superb and stable electrothermal conversion performance.

Solar energy is a high-quality, clean energy source, and photothermal conversion has been extensively studied for solar energy utilization due to its high efficiency [49]. The absorbance and reflectance of a material directly indicate its light absorption capability. In this study, we used a ultraviolet–visible–near-infrared (UV–Vis–NIR) spectrophotometer to obtain the absorption and reflectance spectra of the composites. Figure 6F depicts that the absorbance of CuFC at 800 nm reaches an impressive 25.1, which can be attributed to the stupendous optical nonlinearity and broadband absorption characteristics of CNTs [50]. Moreover, the high-density CNTs are intricately entangled, forming microcavities that effectively trap light and markedly enhance the material’s light absorption performance [51]. However, after the addition of EG, the absorbance of CuFCE-2 slightly decreased, which may be ascribed to the aggregation of larger EG particles on the sample surface. This aggregation could form a weak reflective layer, thereby increasing the material’s reflectance and reducing its light absorption (Fig. 6G).

To evaluate the photothermal conversion capability of the composites, we used infrared light with an optical power density of 160 mW/cm^2^ to simulate solar irradiation and monitored the real-time temperature of the samples applying an FLIR. As shown in Fig. 6H, after 600 s of irradiation under the same light source, the peak temperatures of CuF-0, CuFC-0, CuFE-2, and CuFCE-2 reached 60.1, 78.1, 68.4, and 75.2 °C, respectively, consistent with their absorbance test results. Notably, all composites exhibited a distinct phase change plateau around 40 °C during both heating and cooling, indicating that the PCM within the composites underwent a complete phase change and effectively stored thermal energy. Furthermore, Fig. 6I presents the photothermal conversion curves of CuFCE-2 over 11 cycles. The high degree of consistency among the curves before and after cycling demonstrates the material’s brilliant stability and reliability for practical applications. To summarize, the CuFCE-2 phase change composite reveals extraordinary photothermal conversion performance.

## Conclusion

To sum up, the biomimetic multi-network architecture proposed in this study combines structural innovation with multifunctional performance, allowing composites to effectively tackle both thermal management challenges and electromagnetic radiation issues in advanced electronic systems. CuF was used as the base material, and high-density CNTs tentacles were grown on its surface via chemical vapor deposition to create a biomimetic sea anemone structure. This structure was then combined with a mixture of EG/SEPS/C22 to fabricate the multifunctional phase change composite, CuFCE-2. The synergistic design not only imparts outstanding structural stability but also delivers high thermal conductivity (4.71 W/m·K), superior electrical conductivity (4.29 × 10^6^ S/m), and strong light absorption capability (11.2), which together form the foundation for its versatile energy regulation functionality. In thermal management scenarios, CuFCE-2 significantly reduces the chip operating temperature by 15.7, 34.3, and 60.6 °C under steady-state, short-term, high-load, and transient heat shock conditions, respectively, demonstrating rapid thermal response and effective heat dissipation. Its outstanding EMI shielding ability, with a SE_T_ value reaching 111.1 dB, further ensures reliable operation in complex electronic environments. In addition, the material exhibits stable and efficient electrothermal and photothermal conversion behavior, enabling adaptive thermal control under different energy inputs. Overall, this multifunctional phase change composite offers a comprehensive solution for next-generation thermal interface and shielding materials.

## Materials and Methods

### Materials

The following chemicals were utilized in this study: CuF, with dimensions of 20 mm × 20 mm, a thickness of 5 mm, and a pore size of 95 PPI (pores per inch), was purchased from Kunshan Guangjiayuan Electronic Materials Co. Ltd. (China). Hydrogenated version SEPS was purchased from Dongguan Hetai Plastic Products Co. Ltd. (China). C22 was sourced from Guangdong Tule New Materials Co. Ltd. (China). EG was purchased from Jiangsu Xianfeng Nano Co. Ltd. (China). Hydrochloric acid (HCl; 36% to 38%), TEOS (99.99%), APTES (99%), and sodium hydroxide (NaOH; 98%) were all purchased from Aladdin Chemical Co. Ltd. (China). Ferrocene (99%) and xylene (98%) were purchased from Shanghai MacLean Biochemical Co. Ltd. (China).

### Preparation of CuF-CNTs

Firstly, CuF was added to 10 wt % hydrochloric acid, ultrasonically for 15 min, washed, and dried. Then, acid-washed CuF was added to a mixed solvent of ethanol and water (volume ratio = 4:1), followed by the addition of TEOS and APTES, and sodium hydroxide to adjust the pH to 10, with the reaction stirred for 5 h to obtain F-CuF. A 0.05 g/ml mixed solution of xylene and ferrocene was prepared, placed in a tubular furnace with F-CuF, evacuated, and exposed to hydrogen and argon, with the furnace temperature set to 800 °C and an insulation time of 30 min, resulting in the growth of CNTs on the surface of CuF to obtain CuF-CNTs.

### Preparation of phase change composites

SEPS and EG were added to melted C22 liquid (C22:SEPS:EG = 88:10:2) and mechanically stirred at high speed for 60 min at 90 °C. The resulting mixture was encapsulated in CuF-CNTs to form a composite. The encapsulated composite was hot-pressed at 180 °C and 10 MPa for 20 min to create the final sample, designated as CuFCE-2. Following the same production process: When C22/SEPS/EG (88:10:2) was encapsulated into CuF, the sample was designated as CuFE-2. When C22/SEPS (90:10) was encapsulated into CuF-CNTs, the sample was designated as CuFC-0. When C22/SEPS (90:10) was encapsulated into CuF, the sample was designated as CuF-0.

### Characterization

The microstructure and morphology of CuF, F-CuF, CuF-CNTs, and phase change composites were characterized using FESEM (JEOL SU8010, Japan). EDS (Bruker XFlash, Germany) was coupled to FESEM to investigate elemental mapping in F-CuF. In addition, the details of CuF-CNTs were further observed by TEM (FEI Thermo Talos F200S, Czech Republic). The chemical composition of CuF and CuF-CNTs was analyzed using XPS (Thermo Fisher Escalab 250Xi, UK). All XPS data were calibrated by charge correction. In addition, XRD (PUXI general XD3, China) was used to analyze composites and their raw materials, using Cu–Kα radiation (λ = 0.15406 nm) at a rate of 10°/min and a scanning range of 2θ = 3° to 60°. The chemical composition of composites and their raw materials was analyzed using FTIR (Nicolet IS50, USA). Raman spectra of CuF and CuF-CNTs were obtained using a microscopic confocal Raman spectrometer (LabRAM HR Evolution, HORIBA Jobin Yvon, France).

The thermogravimetric curves of the samples were recorded by a TGA (Mettler-Toledo, Switzerland) under a nitrogen atmosphere from 25 to 600 °C at a heating rate of 10 °C/min. The absorbance and reflectance spectra of the composites were obtained using a UV–Vis–NIR spectrophotometer (UV-3600 plus, SHIMADZU, Japan). The thermal conductivity of the samples was measured using a thermal constant analyzer (TPS500S, Hot Disk AB), and each sample was tested at 3 different points and the average value was taken.

To evaluate the structural stability of the CF-CNTs skeleton and the shape stability of the composite material, structural stability tests and leakage tests were conducted. The structural stability test was carried out as follows: CF-CNTs were immersed in an ethanol solution and subjected to mechanical stirring at 500 rpm and ultrasonic treatment at 160 W, with changes in the samples and solution observed at various time intervals. The structural changes of the tested samples were analyzed using FESEM, and the solution was filtered to examine any detachment of CNTs.

The leakage test involved placing the composite samples in an oven at 60 and 80 °C for a certain period to observe whether any PCM leakage occurred, thereby assessing the shape stability of the composite.

To evaluate the chip thermal management performance of all samples, a ceramic heating element (12 V, 60 W, 20 mm × 20 mm × 2 mm, China) and a DC power supply (MS-305DS, China) were used to simulate chip heating. The chip heating conditions at different power levels and durations were simulated by adjusting the voltage and working time of the DC power supply. The composite was placed on the surface of the simulated chip. The sample temperature was monitored using a FLIR (Therma CAM SC3000), the working temperature of the simulated chip was measured with a thermocouple, and the data were collected using an Agilent data acquisition system (34970A).

The EMI SE was tested by Vector Network Analyzer (Agilent E5071C) over the X-band (8.2 to 12.4 GHz), and the tested samples were asked to cut to the dimension of 22.9 mm × 10.2 mm × 2 mm.

The electrothermal conversion test employs a regulated DC power supply, with the tested sample connected to the power supply to form a closed loop via a wire. A FLIR records the sample’s temperature change curve over time.

The photothermal conversion simulation experiment was used to explore the photothermal conversion performance of the composite, which consisted of a power-adjustable lamp (working power: 0 to 275 W) to simulate the solar light source, and a FLIR to collect the temperature changes over time. The light intensity of the simulated light source was measured using an optical power density meter (CEL-NP2000).

## Data Availability

All data are available in the main text or the Supplementary Materials.
