# Supplementary material for: Sea Anemone-Inspired Phase Change Composites for Efficient Heat Dissipation and Ultra-High Electromagnetic Interference Shielding
Source: Research (Wash D C). 2026 Jan 12;9:1075. doi: 10.34133/research.1075 (PMC12794197; doi:10.34133/research.1075)
Supplement: Supplementary 1 — Supplementary Text Tables S1 to S4 Figs. S1 to S4 [file research.1075.f1.docx]

**Supplementary material**

**Sea Anemone-inspired Phase Change Composites for Efficient Heat Dissipation and Ultra-high Electromagnetic Interference Shielding**

Xiaoling He ^1,2,^ ^‡^, Wenjian Zhang ^1, ‡^, Tao Liu ^1^, Qianhui Lin ^2^, Zexi Zhang ^1^, Ying Chen ^2^, Jiye Luo ^3 *^, Chengqiang Cui ^1^, Xinxin Sheng ^2 *^

^1^ *State Key Laboratory of Precision Electronic Manufacturing Technology and Equipment, School of Electromechanical Engineering, Guangdong University of Technology, Guangzhou 510006, China.*

^2^ *Guangdong Provincial Key Laboratory of Functional Soft Condensed Matter, School of Materials and Energy, Guangdong University of Technology, Guangzhou 510006, China.*

^3^ *School of Chemical Engineering and Light Industry, Guangdong University of Technology, Guangzhou 510006, China.*

^‡^ Xiaoling He and Wenjian Zhang contributed equally to this work.

^*^ Address correspondence to: luojiye@gdut.edu.cn (J. Luo), xinxin.sheng@gdut.edu.cn (X. Sheng).

**2. Experimental sections**

**2.1. Modeling and thermal behavior simulation of phase change composites**

The thermal simulation framework was developed by first constructing a 3D interconnected CuF model using CINEMA 4D software (**Fig.6b**), from which a representative section was selected and processed in R2V software to generate a 2D cross-sectional view. This 2D profile was then imported into CAD and arrayed to create a 2×2 mm CuF skeleton structure. By computationally optional filling this skeleton with C22, EG, and CNTs, we obtained the 2D thermal simulation models of CuF-0, CuFC-0, CuFE-2, and CuFCE-2 composites. These multiphase systems exhibit fundamentally distinct heat transfer characteristics from homogeneous materials due to component synergies, where thermal conductivity depends not only on constituent thermophysical properties but also on phase spatial distribution, interfacial bonding, and scale effects. To accurately model these phenomena, finite element analysis (COMSOL Multiphysics) was employed to establish a phase-change heat transfer-solid-fluid coupling model for simulating the thermal behavior of different composite materials. The relevant simulation parameters are listed in **Tab. S1** and **Tab. S2**. The boundary conditions are set to bottom heating of 1 W, three-sided convection cooling, and an initial temperature of 25°C, which is controlled by the following equation:

**(1)** Energy conservation equation

$$\rho c_{p}\frac{\partial T}{\partial t}+\rho c_{p}u\cdot\nabla T+\nabla\cdot\left( \dot{q}_{c}+\dot{q}_{r} \right)=\beta T\left( \frac{\partial p}{\partial t}+u\cdot\nabla p \right)+\tau:\nabla u+Q$$

The paraffin wrapped in the composite phase change material hardly flows after melting, so the fluid flow term is ignored and simplified to:

$$\rho c_{p}\frac{\partial T}{\partial t}=\nabla\cdot\left( \lambda\nabla T \right)+Q$$

Where  ρ represents the density,  c_p_ is the specific heat capacity,  λ is the thermal conductivity, and  ∇T is the temperature gradient.  Q denotes the latent heat power density released or absorbed per unit volume during the phase change, which is expressed as：

$$Q=\rho\cdot L\cdot D(T)\cdot\frac{\partial T}{\partial t}$$

Where L is the latent heat (J/kg), and D(T) is a Gaussian distribution function describing the phase change behavior.

**(2)** Thermal conductivity-melting fraction coupling equation

$$\lambda\left( T \right)=\lambda_{s}+\left( \lambda_{l}-\lambda_{s} \right)\cdot\varphi(T)$$

Among them, the solid-phase thermal conductivity (λ_s_) and liquid-phase thermal conductivity (λ_l_) of paraffin are defined based on its material properties, while the thermal conductivities of copper foam and graphite carbon nanotubes are set as constants.

**(3)** Phase change range control

$$\varphi\left( T \right)=\left\{ \begin{aligned} 0 T＜T_{pc}-\frac{\Delta T_{pc}}{2} \\ \frac{T-(T_{pc}-\frac{\Delta T_{pc}}{2})}{\Delta T_{pc}} T_{pc}-\frac{\Delta T_{pc}}{2}＜T＜T_{pc}+\frac{\Delta T_{pc}}{2} \\ 1 T＞T_{pc}+\frac{\Delta T_{pc}}{2} \end{aligned} \right.$$

Thermal conductivity coupling λ(T) and density coupling ρ(T) depend on φ(T) to realize the transition of physical property parameters.

**(4)** Specific heat capacity model equation

$$c_{p}(T)=c_{p,s}+\varphi(T)\cdot\left( c_{p,l}-c_{p,s} \right)+L\cdot D(T)$$

Where the Gaussian function D(T) defines the temperature distribution of latent heat release:

$$D\left( T \right)=\frac{e^{-\frac{{(T-T_{pc})}^{2}}{{(\frac{\Delta T_{pc}}{4})}^{2}}}}{\sqrt{\pi{(\frac{\Delta T_{pc}}{4})}^{2}}}$$

Additionally, two fundamental assumptions were adopted in the thermal simulations to simplify computations. Firstly, all material components were considered isotropic in their thermophysical properties. Secondly, the electronic conductivity, thermal conductivity, specific heat capacity, and density of all the composite materials were assumed temperature-invariant during heating processes.

In **Fig. S2**, the simulation results demonstrate excellent agreement with experimental data, confirming that the computational model accurately captures the experimentally observed thermodynamic behavior. This validates the accuracy of the heat transfer model, boundary conditions, and parameter settings used in the simulation.

**Tab. S1** Thermal conductivity related parameters.

| Parameter | SEPS/C22 | Cu | EG | CNTs |
| --- | --- | --- | --- | --- |
| Thermal conductivity  W/(m∙K) | 0.375 | 380 | 150 | 3000 |
| Density  kg/m^3^ | 928 | 8900 | 1100 | 1500 |
| Heat capacity  J/(kg∙K) | 1925/2200 | 385 | 710 | 675 |

**Tab. S2** Phase change material related parameters

| Parameter | SEPS/C22 |
| --- | --- |
| Phase transition temperature (K) | 316.55 |
| Phase change temperature range (K) | 6 |
| Fuzzy zone constant (kg/(m^3^∙s)) | 100000 |
| Solid specific heat (J/(kg∙K)) | 1925 |
| Liquid specific heat J/(kg∙K) | 2200 |
| Latent heat (J/kg) | 96560 |
| Thermal volume expansion coefficient (1/K) | 0.0001 |


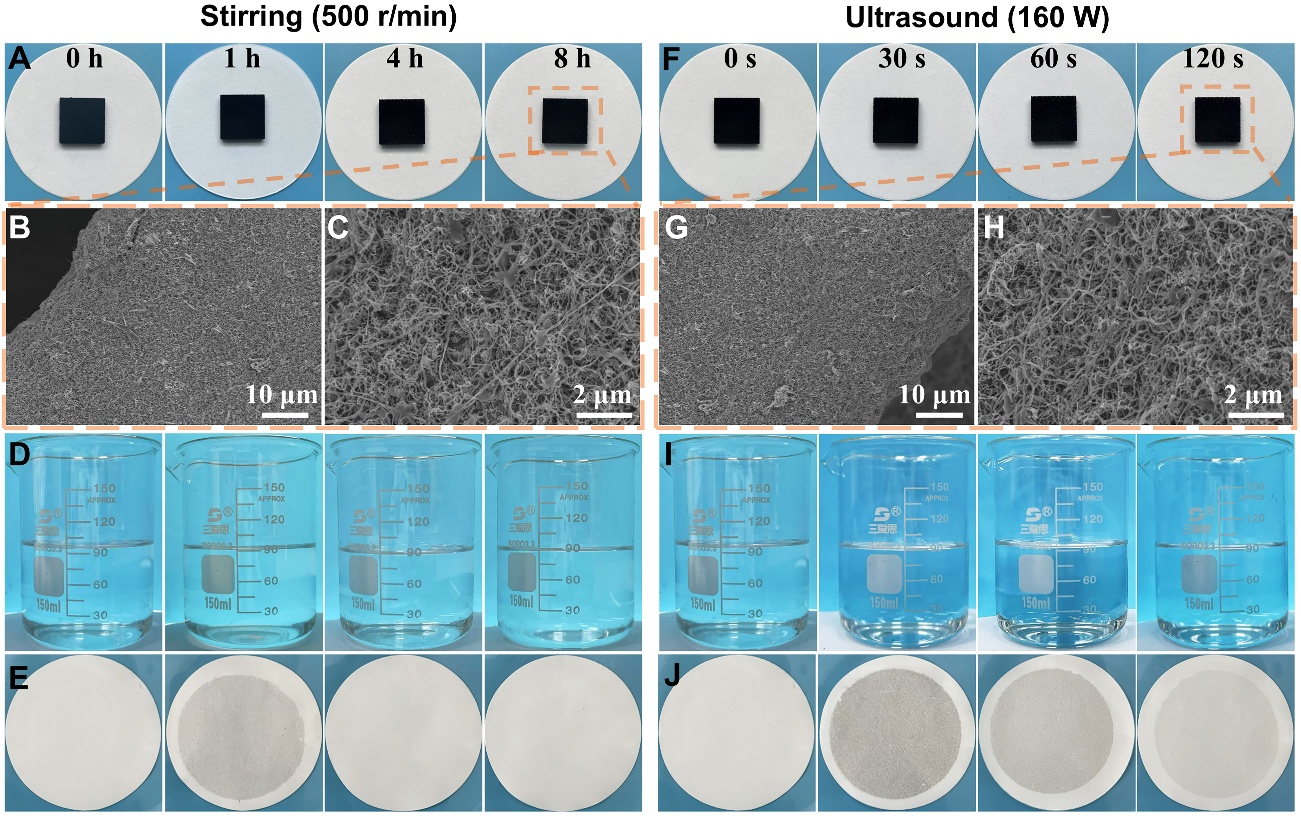


**Fig. S1** Characterization of the structural stability of CuF-CNTs. (A) Digital photos of CuF-CNTs after stirring at 500 r/min; (B) and (C) are FESEM of CuF-CNTs after stirring for 8 h; (D) is the solution of the stirred CuF-CNTs; (E) Filter paper after the (D) solution was filtered; (F) Digital photos of CuF-CNTs after ultrasound at 160 W; (G) and (H) are FESEM of CuF-CNTs under ultrasound at 120 s; (I) is the solution of the ultrasound CuF-CNTs; (J) Filter paper after the (I) solution was filtered.


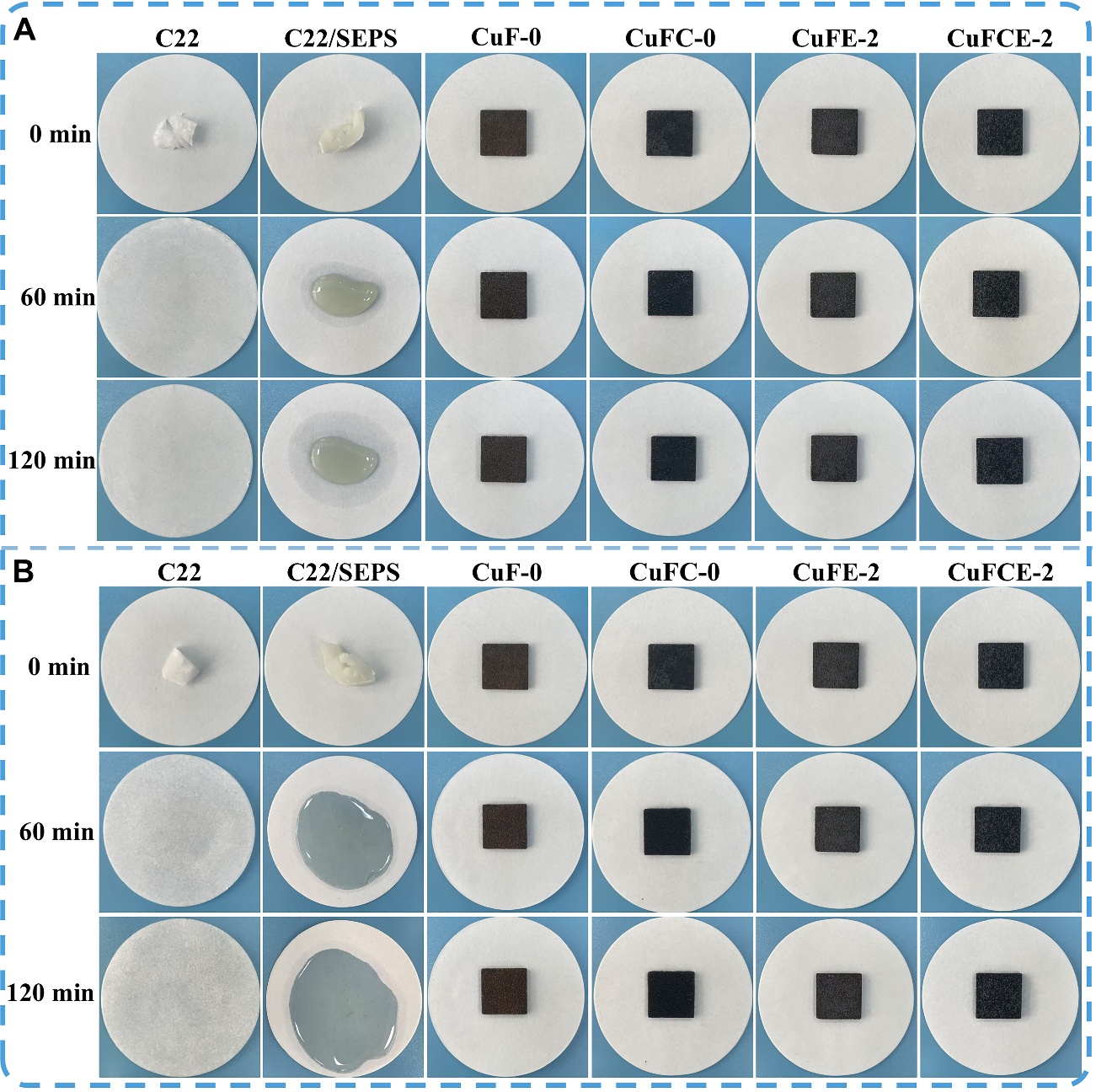


**Fig. S2** Characterization of the shape stability of phase change composite (A) Digital image of composite leakage test at 60°C; (B) Digital image of composite material leakage test at 80°C.


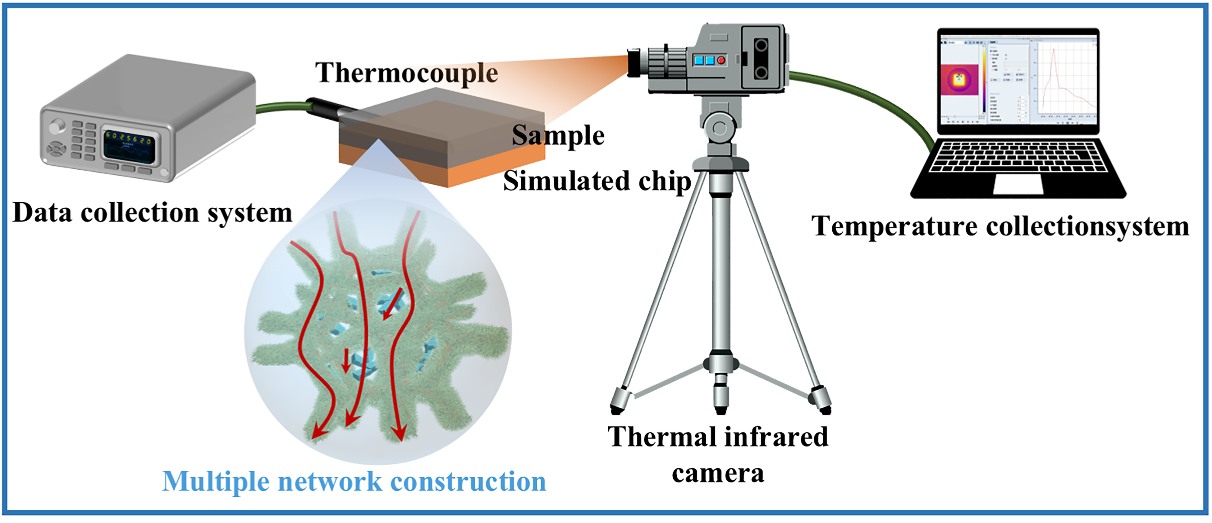


**Fig. S3** A measurement platform for evaluating the thermal management performance.


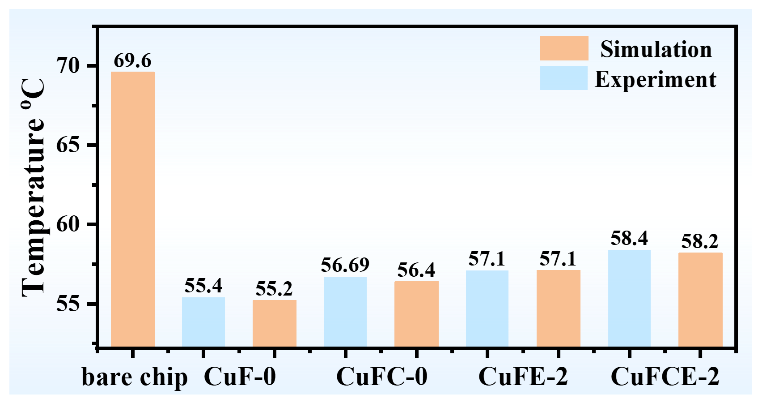


**Fig. S4** Comparison of simulation results with experimental results.


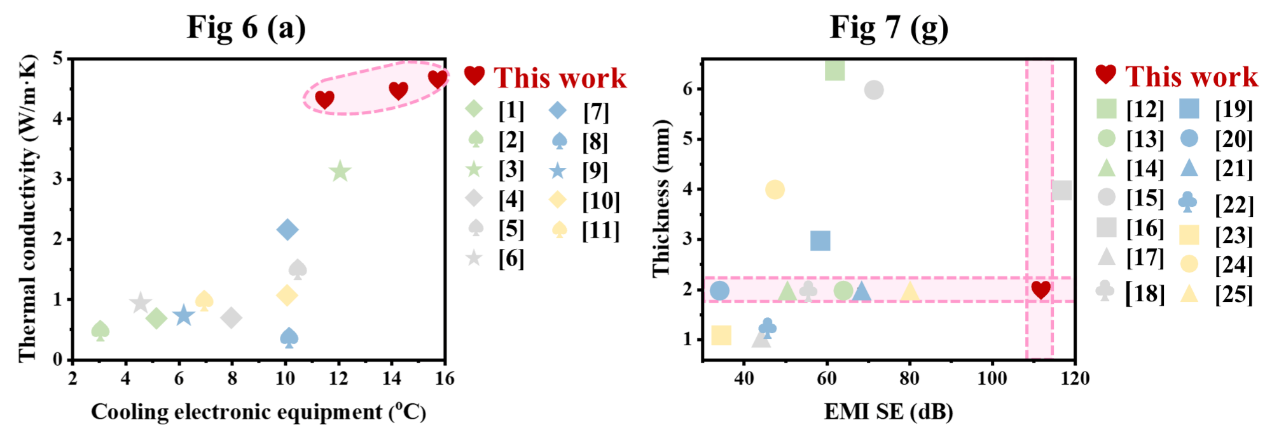


**Tab. S3** References and parameters in **Fig. 6**a

| References | Thermal conductivity (W/m·K) | Cooling electronic equipment (°C) |
| --- | --- | --- |
| [1] | 0.68 | 5.1 |
| [2] | 0.510 | 2.8 |
| [3] | 3.15 | 12 |
| [4] | 0.701 | 7.9 |
| [5] | 1.53 | 10.4 |
| [6] | 0.94 | 4.5 |
| [7] | 2.16 | 10 |
| [8] | 0.394 | 10.1 |
| [9] | 0.76 | 6.1 |
| [10] | 1.08 | 10 |
| [11] | 0.97 | 6.9 |


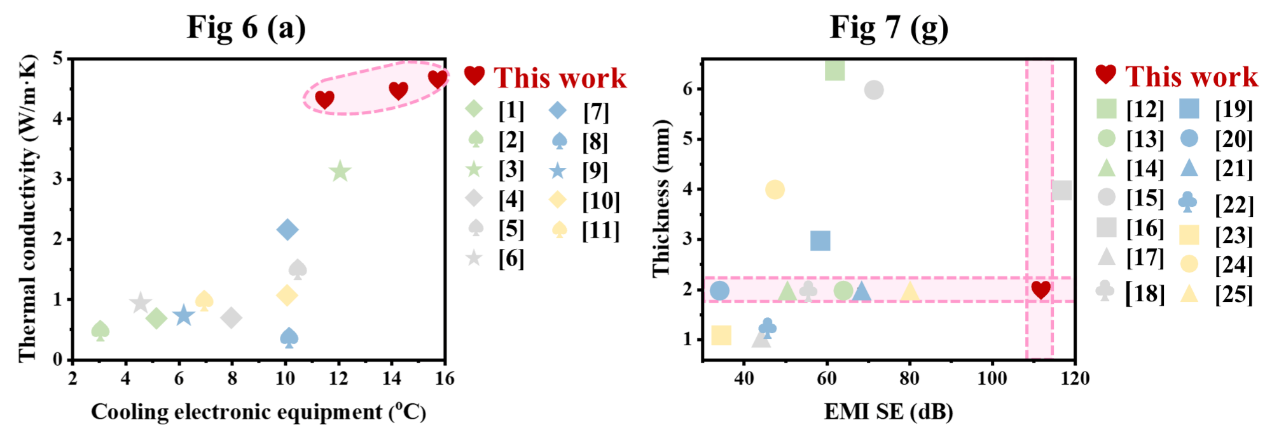


**Tab. S4** References and parameters in **Fig. 7**g

| References | EMI SE (dB) | Thickness (nn) |
| --- | --- | --- |
| [12] | 61.5 | 6.5 |
| [13] | 63.8 | 2 |
| [14] | 50.3 | 2 |
| [15] | 71.08 | 6 |
| [16] | 116.7 | 4 |
| [17] | 44.2 | 1 |
| [18] | 55.3 | 2 |
| [19] | 57.93 | 3 |
| [20] | 33.7 | 2 |
| [21] | 67.86 | 2 |
| [22] | 44.7 | 1.2 |
| [23] | 34.12 | 1.1 |
| [24] | 47.1 | 4 |
| [25] | 79.87 | 2 |

**References:**

1. Xie Y, Xiao S, Chen W, Hu X, Liu Y, Jiang L, Luo L, Luo W, Ma Y, Jiang X, He Y, Li Q. Shape-stabilized nanosilver-modified grapefruit peel-based porous carbon composite phase change material with high thermal conductivity, photothermal conversion performance and thermal management capability. *J Energy Storage*. 2024;83: 110819.

2. Tan J, Zhu G, Yang F, Zhang S, Wu Q, Xu L, Li Y, Tan L, Meng X, Yu J, Li L. Multi-scale-filler reinforcement strategy enabled stretchable silicone elastomer with synergistically enhanced thermal conductivity and mechanical strength. *Composites Part A: Applied Science and Manufacturing*. 2023;175: 107784.

3. He X, Zhang W, Yang Y, Yang G, Zhang Y, Huang G, Luo J, Cui C, Sheng X. Triple-network structured phase change composite based on “rod-brush” CNTs-CFs with high thermal conductivity. *Compos Sci Technol.* 2025;262: 111080.

4. Yin G, López AM, Collado I, Vázquez-López A, Ao X, Hobson J, Prolongo SG, Wang D. MXene multi-functionalization of polyrotaxane based PCMs and the applications in electronic devices thermal management. *Nano Materials Science*. 2024;6: 495–503.

5. An D, Chen Y, He R, Yu H, Sun Z, Liu Y, Liu Y, Lian Q, Feng W, Wong C. The polymer-based thermal interface materials with improved thermal conductivity, compression resilience, and electromagnetic interference shielding performance by introducing uniformly melamine foam. *Adv Compos Hybrid Mater*. 2023;6: 136.

6. Wang S, Huang Q, Sun Z, Wang Y, Liang T, Wang B, Fan C, Liu C. Porous carbon network-based composite phase change materials with heat storage capacity and thermal management functions. *Carbon*. 2024;226: 119174.

7. Jiang W, Wang R, Zhu T, Feng M, Sun D, Yang J, Qi X, Wang Y. Tree-ring structured phase change materials with high through-plane thermal conductivity and flexibility for advanced thermal management. *Chemical Engineering Journal*. 2024;479: 147622.

8. Zhang W, Pan R, Yang J, Liu M, Yao Y, Zhang A, Gong Y, Gan Z, Hu R, Ding J, Chen L, Zhang X, Xue M, Tian X. Polystyrene shell-based “coconut-like” and “pomegranate-like” microencapsulated phase change materials: Formation mechanism, thermal conductivity/stability enhancement and their application in thermal management. *Chemical Engineering Journal*. 2024;498: 155758.

9. Miaari AA, Mohaisen KO, Lawag RA, Al-Ahmed A, Ali HM. Characterization of a novel sustainable shape stabilized phase change material based on oil ash for photovoltaic thermal management. *Solar Energy*. 2025;292: 113455.

10. Yan Y, Wu B, Qian G, Lan H, Alam MM, Xia L, Qian J. Ultra-wideband electromagnetic interference shielding effectiveness composite with elevated thermal conductivity. *Composites Part A: Applied Science and Manufacturing*. 2023;167: 107430.

11. Zhang W, He X, Huang G, Zhang Y, Yang G, Cui C, Sheng X. Dual-oriented elastic composites based on novel CF-CNTs fillers with super thermal management capabilities. *Appl Mater Today*. 2025;44: 102757.

12. Habibpour S, Rahimi-Darestani Y, Salari M, Zarshenas K, Taromsari SM, Tan Z, Hamidinejad M, Park CB, Yu A. Synergistic Layered Design of Aerogel Nanocomposite of Graphene Nanoribbon/MXene with Tunable Absorption Dominated Electromagnetic Interference Shielding. *Small*. 2024;20: 2404876.

13. Wei Z, Cai Y, Zhan Y, Meng Y, Pan N, Jiang X, Xia H. Ultra-Low Loading of Ultra-Small Fe3O4 Nanoparticles on Nonmodified CNTs to Improve Green EMI Shielding Capability of Rubber Composites. *Small*. 2024;20: 2307148.

14. Zhang Y-C, Ding R, Su P-G, Zeng F-R, Jia X-X, Hu Z-Y, Wang Y-Z, Zhao H-B. Biomimetic Ambient-Pressure-Dried Aerogels with Oriented Microstructures for Enhanced Electromagnetic Shielding. *Adv Funct Mater*. 2025;35: 2414683.

15. Wu C, Zeng L, Chang G, Zhou Y, Yan K, Xie L, Xue B, Zheng Q. Composite phase change materials embedded into cellulose/polyacrylamide/graphene nanosheets/silver nanowire hybrid aerogels simultaneously with effective thermal management and anisotropic electromagnetic interference shielding. *Adv Compos Hybrid Mater*. 2023;6: 31.

16. Hu B, Guo H, Li T, Cao X, Cao M, Qi W, Cui Y, Li B. Engineering tiramisu-like phase change nanocomposite for superior thermal energy management and electromagnetic interference shielding. *Journal of Materials Science & Technology*. 2025;206: 113–124.

17. Ouyang W, Mei L, Liu Q, Ding C, Liu Y, Zhao C, Xu L, Lu F, Luo D, Miao C, Bai Y, Lu Q, Luo T, Wu Z. Ultrathin-flexible multifunctional MXene composite hydrogels with good mechanical properties-high strain sensitivity and ultra-broadband EMI shielding performances. *Chemical Engineering Journal*. 2024;494: 153068.

18. Yao J, Zhou J, Peng G, An D, Yao Z. Exoskeleton-like mechanical enhanced low-density aerogel with electromagnetic interface shielding and infrared stealth. *Composites Part A: Applied Science and Manufacturing*. 2024;177: 107954.

19. Li Y, Diao X, Li P, Liu P, Gao Y, Zhao Z, Chen X, Wang G. Advanced multifunctional Co/N co-doped carbon foam-based phase change materials for wearable thermal management. *Chem Eng J*. 2024;485: 149858.

20. Xu L, Si R, Ni Q, Chen J, Zhang J, Ni Q-Q. Synergistic magnetic/dielectric loss and layered structural design of Ni@carbon fiber/Ag@graphene fiber/polydimethylsiloxane composite for high-absorption EMI shielding. *Carbon*. 2024;225: 119155.

21. Guo Q, Tian H, Cheng Y, Wang S, Li Z, Hao H, Liu J, Jiao K, Gao X, Zhang J. Structural–Functional Integrated Graphene-Skinned Aramid Fibers for Electromagnetic Interference Shielding. *ACS Nano*. 2024;18: 33566–33575.

22. Chen M, Li M, Gao Y, He S, Zhan J, Zhang K, Huo Y, Zhu J, Zhou H, Fan J, Chen R, Wang H. Flexible and Robust Core–Shell PANI/PVDF@PANI Nanofiber Membrane for High-Performance Electromagnetic Interference Shielding. *Nano Lett*. 2024;24: 2643–2651.

23. Yue H, Ou Y, Wang J, Wang H, Du Z, Du X, Cheng X. Ti3C2Tx MXene/delignified wood supported flame-retardant phase-change composites with superior solar-thermal conversion efficiency and highly electromagnetic interference shielding for efficient thermal management. *Energy*. 2024;286: 129441.

24. Wang S, Wang Z, Zheng SY, Yang J. Multifunctional heterostructured composite foam with tunable electromagnetic interference shielding. *Composites Science and Technology*. 2024;248: 110482.

25. Guo Y, Zhang Y, Wu H, Guo S. Gradient distribution of segregated conductive network in polyvinylidene fluoride nanocomposites to achieve outstanding electromagnetic interference shielding with low reflection. *Journal of Materials Science & Technology*. 2024;190: 24–32.
